# Supplementary material for: Predation and fragmentation portrayed in the statistical structure of prey time series
Source: BMC Ecol. 2009 May 6;9:10. doi: 10.1186/1472-6785-9-10 (PMC2689204; doi:10.1186/1472-6785-9-10)
Supplement: Additional file 2 — Voles and related classes ODDox Documentation. ODDox documentation of the agent-based model (ALMaSS) applied by Hendrichsen et al. The documentation is started by activating main.html. [file 1472-6785-9-10-S2.zip › Vole_ODDox/tov__declaration_8h.html]

ALMaSS ODDox: tov\_declaration.h File Reference

- Main Page
- Related Pages
- Classes
- Files

# tov\_declaration.h File Reference

|  |  |
| --- | --- |
|  |  |
| Enumerations | |
| enum | TTypesOfVegetation {     tov\_Carrots = 0, tov\_CloverGrassGrazed1, tov\_CloverGrassGrazed2, tov\_FieldPeas,     tov\_FodderBeet, tov\_Maize, tov\_NaturalGrass, tov\_NoGrowth,     tov\_None, tov\_Oats, tov\_OBarleyPeaCloverGrass, tov\_OCarrots,     tov\_OCloverGrassGrazed1, tov\_OCloverGrassGrazed2, tov\_OCloverGrassSilage1, tov\_OFieldPeas,     tov\_OFirstYearDanger, tov\_OGrazingPigs, tov\_OOats, tov\_OPermanentGrassGrazed,     tov\_OPotatoes, tov\_OSeedGrass1, tov\_OSeedGrass2, tov\_OSetaside,     tov\_OSpringBarley, tov\_OSpringBarleyClover, tov\_OSpringBarleyGrass, tov\_OSpringBarleyPigs,     tov\_OTriticale, tov\_OWinterBarley, tov\_OWinterRape, tov\_OWinterRye,     tov\_OWinterWheatUndersown, tov\_PermanentGrassGrazed, tov\_PermanentGrassLowGrazed, tov\_PermanentSetaside,     tov\_Potatoes, tov\_PotatoesIndustry, tov\_SeedGrass1, tov\_SeedGrass2,     tov\_Setaside, tov\_SpringBarley, tov\_SpringBarleyCloverGrass, tov\_SpringBarleyGrass,     tov\_SpringBarleySeed, tov\_SpringBarleySilage, tov\_SpringRape, tov\_SpringWheat,     tov\_Triticale, tov\_WinterBarley, tov\_WinterRape, tov\_WinterRye,     tov\_WinterWheat, tov\_WWheatPControl, tov\_WWheatPToxicControl, tov\_WWheatPTreatment,     tov\_AgroChemIndustryCereal, tov\_WinterWheatShort, tov\_Orchard, tov\_OSBarleySilage,     tov\_OFieldPeasSilage, tov\_FieldPeasStrigling, tov\_MaizeStrigling, tov\_SpringBarleyStrigling,     tov\_SpringBarleyCloverGrassStrigling, tov\_WinterBarleyStrigling, tov\_WinterRapeStrigling, tov\_WinterRyeStrigling,     tov\_WinterWheatStrigling, tov\_SpringBarleyPeaCloverGrassStrigling, tov\_YoungForest, tov\_SpringBarleyStriglingSingle,     tov\_SpringBarleyStriglingCulm, tov\_WinterWheatStriglingSingle, tov\_WinterWheatStriglingCulm, tov\_Undefined   } |

---

## Enumeration Type Documentation

enum TTypesOfVegetation

**Enumerator:**
:   |  |  |
    | --- | --- |
    | *tov\_Carrots* |  |
    | *tov\_CloverGrassGrazed1* |  |
    | *tov\_CloverGrassGrazed2* |  |
    | *tov\_FieldPeas* |  |
    | *tov\_FodderBeet* |  |
    | *tov\_Maize* |  |
    | *tov\_NaturalGrass* |  |
    | *tov\_NoGrowth* |  |
    | *tov\_None* |  |
    | *tov\_Oats* |  |
    | *tov\_OBarleyPeaCloverGrass* |  |
    | *tov\_OCarrots* |  |
    | *tov\_OCloverGrassGrazed1* |  |
    | *tov\_OCloverGrassGrazed2* |  |
    | *tov\_OCloverGrassSilage1* |  |
    | *tov\_OFieldPeas* |  |
    | *tov\_OFirstYearDanger* |  |
    | *tov\_OGrazingPigs* |  |
    | *tov\_OOats* |  |
    | *tov\_OPermanentGrassGrazed* |  |
    | *tov\_OPotatoes* |  |
    | *tov\_OSeedGrass1* |  |
    | *tov\_OSeedGrass2* |  |
    | *tov\_OSetaside* |  |
    | *tov\_OSpringBarley* |  |
    | *tov\_OSpringBarleyClover* |  |
    | *tov\_OSpringBarleyGrass* |  |
    | *tov\_OSpringBarleyPigs* |  |
    | *tov\_OTriticale* |  |
    | *tov\_OWinterBarley* |  |
    | *tov\_OWinterRape* |  |
    | *tov\_OWinterRye* |  |
    | *tov\_OWinterWheatUndersown* |  |
    | *tov\_PermanentGrassGrazed* |  |
    | *tov\_PermanentGrassLowGrazed* |  |
    | *tov\_PermanentSetaside* |  |
    | *tov\_Potatoes* |  |
    | *tov\_PotatoesIndustry* |  |
    | *tov\_SeedGrass1* |  |
    | *tov\_SeedGrass2* |  |
    | *tov\_Setaside* |  |
    | *tov\_SpringBarley* |  |
    | *tov\_SpringBarleyCloverGrass* |  |
    | *tov\_SpringBarleyGrass* |  |
    | *tov\_SpringBarleySeed* |  |
    | *tov\_SpringBarleySilage* |  |
    | *tov\_SpringRape* |  |
    | *tov\_SpringWheat* |  |
    | *tov\_Triticale* |  |
    | *tov\_WinterBarley* |  |
    | *tov\_WinterRape* |  |
    | *tov\_WinterRye* |  |
    | *tov\_WinterWheat* |  |
    | *tov\_WWheatPControl* |  |
    | *tov\_WWheatPToxicControl* |  |
    | *tov\_WWheatPTreatment* |  |
    | *tov\_AgroChemIndustryCereal* |  |
    | *tov\_WinterWheatShort* |  |
    | *tov\_Orchard* |  |
    | *tov\_OSBarleySilage* |  |
    | *tov\_OFieldPeasSilage* |  |
    | *tov\_FieldPeasStrigling* |  |
    | *tov\_MaizeStrigling* |  |
    | *tov\_SpringBarleyStrigling* |  |
    | *tov\_SpringBarleyCloverGrassStrigling* |  |
    | *tov\_WinterBarleyStrigling* |  |
    | *tov\_WinterRapeStrigling* |  |
    | *tov\_WinterRyeStrigling* |  |
    | *tov\_WinterWheatStrigling* |  |
    | *tov\_SpringBarleyPeaCloverGrassStrigling* |  |
    | *tov\_YoungForest* |  |
    | *tov\_SpringBarleyStriglingSingle* |  |
    | *tov\_SpringBarleyStriglingCulm* |  |
    | *tov\_WinterWheatStriglingSingle* |  |
    | *tov\_WinterWheatStriglingCulm* |  |
    | *tov\_Undefined* |  |

```
00007              {
00008   tov_Carrots = 0, tov_CloverGrassGrazed1, // 1
00009        tov_CloverGrassGrazed2, // 2
00010        tov_FieldPeas, // 3
00011        tov_FodderBeet, // 4
00012        tov_Maize, // 5
00013        tov_NaturalGrass, // 6
00014        tov_NoGrowth, // 7
00015        tov_None, // 8
00016        tov_Oats, // 9
00017        tov_OBarleyPeaCloverGrass, // 10
00018 
00019        tov_OCarrots, tov_OCloverGrassGrazed1, tov_OCloverGrassGrazed2, tov_OCloverGrassSilage1, tov_OFieldPeas,
00020        tov_OFirstYearDanger, tov_OGrazingPigs, tov_OOats, tov_OPermanentGrassGrazed, tov_OPotatoes, // 20
00021 
00022        tov_OSeedGrass1, tov_OSeedGrass2, tov_OSetaside, tov_OSpringBarley, tov_OSpringBarleyClover, tov_OSpringBarleyGrass,
00023        tov_OSpringBarleyPigs, tov_OTriticale, tov_OWinterBarley, tov_OWinterRape, // 30
00024 
00025        tov_OWinterRye, tov_OWinterWheatUndersown, tov_PermanentGrassGrazed, tov_PermanentGrassLowGrazed, tov_PermanentSetaside,
00026        tov_Potatoes, tov_PotatoesIndustry, tov_SeedGrass1, tov_SeedGrass2, tov_Setaside, // 40
00027 
00028        tov_SpringBarley, tov_SpringBarleyCloverGrass, tov_SpringBarleyGrass, tov_SpringBarleySeed, tov_SpringBarleySilage,
00029        tov_SpringRape, tov_SpringWheat, tov_Triticale, tov_WinterBarley, tov_WinterRape, // 50
00030 
00031        tov_WinterRye, tov_WinterWheat, tov_WWheatPControl, tov_WWheatPToxicControl, tov_WWheatPTreatment, tov_AgroChemIndustryCereal, //56
00032        tov_WinterWheatShort, tov_Orchard, tov_OSBarleySilage, tov_OFieldPeasSilage, // 60
00033 
00034        tov_FieldPeasStrigling, tov_MaizeStrigling, tov_SpringBarleyStrigling, tov_SpringBarleyCloverGrassStrigling,
00035        tov_WinterBarleyStrigling, tov_WinterRapeStrigling, tov_WinterRyeStrigling, tov_WinterWheatStrigling, //68
00036        tov_SpringBarleyPeaCloverGrassStrigling, tov_YoungForest,
00037 
00038        tov_SpringBarleyStriglingSingle, tov_SpringBarleyStriglingCulm, tov_WinterWheatStriglingSingle,
00039        tov_WinterWheatStriglingCulm,
00040 
00041 
00042        tov_Undefined // Must be in here and the last one as well!
00043 }
```

---

Generated on Thu Jan 22 14:13:45 2009 for ALMaSS ODDox by 
 1.5.6
